# p53: A Regulator of Ferroptosis Induced by Galectin-1 Derived Peptide 3 in MH7A Cells

**DOI:** 10.3389/fgene.2022.920273

**Published:** 2022-07-04

**Authors:** Junzheng Hu, Rui Zhang, Qing Chang, Mingliang Ji, Haixiang Zhang, Rui Geng, Chao Li, Zhen Wang

**Affiliations:** ^1^ Department of Orthopaedics, Zhongda Hospital, Southeast University, Nanjing, China; ^2^ Department of Orthopaedics, The First Hospital Affiliated to China Pharmaceutical University, Nanjing, China

**Keywords:** bioactive peptide, MH7A, ferroptosis, p53, synovial fibroblast, ra

## Abstract

**Backgrounds:** Rheumatoid arthritis synovial fibroblasts (RASFs) are the primary cells responsible for destruction of marginal cartilage in rheumatoid arthritis (RA). G1dP3, a bioactive peptide derived from galectin-1 domain, possesses potent anti-inflammatory and anti-proliferation properties in RASFs. This study aimed to determine the effects of G1dP3 ferroptosis induction in RASFs and to further clarify the possible mechanisms.

**Methods:** TNF-α was used to establish a RA model in MH7A cells. Cell Counting Kit-8 assays were employed to detect MH7A cell viability with different treatments. The occurrence of ferroptosis was examined by Lipid ROS assay, cellular labile iron pool measurement, reduced glutathione/oxidized glutathione activity, Gpx4 expression and transmission electron microscopy (TEM) morphology observation. Lentiviral-mediated siRNA interference was used to determine the downstream pathway.

**Results:** G1dP3 markedly suppressed MH7A cell viability induced by TNF-α. G1dP3-treated MH7A cells presented the morphological features of ferroptosis. Moreover, G1dP3 triggered ferroptosis in MH7A cells by promoting the accumulation of lipid peroxides as well as iron deposition. Inhibition of ferroptosis alleviated G1dP3-mediated suppression of MH7A cell viability. Furthermore, G1dP3 increased p53 expression, which in turn transcriptionally suppressed SLC7A11, a key component of system X_c_^−^ essential for ferroptosis. Knockdown of p53 abrogated the ferroptotic effects of G1dP3 on MH7A cells.

**Conclusion:** Our findings reveal that the bioactive peptide G1dP3 promotes RASFs ferroptosis cell death via a p53/SLC7A11 axis-dependent mechanism, suggesting its potential role in the treatment of RA.

## Introduction

Rheumatoid arthritis (RA) is a common complex systemic disease, afflicting 0.5–1% of populations worldwide (Smolen et al., 2016). This disease is mainly characterized by synovial membrane inflammation and disruption of articular cartilage (Aletaha et al., 2011). It has been reported that synovial fibroblasts are the effector cells of inflammatory responses in RA (Klippel, 2000; Weyand and Goronzy, 2021). Thus, inhibiting synovial fibroblast activation is a potential strategy for RA prevention and treatment.

As a new branch of proteomics, peptidomics can be used to analyze endogenous protein fragments in body fluids and tissues (Baggerman et al., 2005). Biologically active peptides could be a potential biomarker for RA diagnosis, and attenuated inflammation both *in vitro* and *in vivo* (Roark et al., 2016; Smolen, 2020). From a perspective of peptidomics, differentially expressed peptides have been identified between RA and normal synovial tissue groups in our previous study (8). In particular, G1dP3, a galectin-1 derived peptide with two distinct polar/basic residues and two distinct polar/acid residues, could significantly decrease the expression levels of inflammation cytokines (e.g., IL-6, IL-1β, MMP-13, and MMP-1) in MH7A synovial cells induced by TNF-α. In addition, G1dP3 exhibits a proapoptotic effect on MH7A cells, implying its great potential for RA treatment (Hu et al., 2020). However, the underlying mechanism of G1dP3 in inhibiting MH7A cell viability remains unclear.

Ferroptosis is a cell death modality regulated by iron overload and reactive oxygen species (ROS) production (Dixon et al., 2012; Cao and Dixon, 2016). Mechanistic studies have shown that ferroptosis occurs when lipid peroxidation or iron metabolism is dysregulated, together with glutathione (GSH) deletion and glutathione peroxidase (Gpx4) inactivation (Dixon et al., 2012; Cao and Dixon, 2016). Thus, ferroptosis can be induced by decreasing glutamate-cysteine ligase, inhibiting cystine/glutamate antiporter xCT (SLC7A11/system X_c_^−^), suppressing Gpx4 activity, scavenging free radical (Ferrostatin-1), promoting iron chelation and blocking Fenton reactions (deferoxamine, DFO) (Dixon et al., 2012; Cao and Dixon, 2016; Xie et al., 2016). To our knowledge, the role of ferroptosis in RA synovial fibroblasts has not been clarified, and whether the anti-proliferation properties of G1dP3 are related to ferroptosis in RA synovial fibroblasts remains to be explored.

P53, the most commonly mutated tumour suppressor gene, has been testified to play a dual role in ferroptosis. On the one hand, p53 can induce ferroptosis by upregulating GLS2 and SAT1 expression or downregulating SLC7A11 expression. On the other hand, p53 can suppress ferroptosis by increasing CDKN1A expression or reducing DPP4 activity (Jiang et al., 2015a; Wang et al., 2016; Xie et al., 2017). p53 induces ROS-regulated ferroptosis through GSH depletion caused by xCT downregulation. Besides, SLC7A11 can function a transcriptional target for p53 to bind to its promoter region, which facilitates the cellullar uptake of extracellular cysteine, and the intracellular cystine is coverted to cysteine prior to GSH synthesis (Dixon et al., 2012; Guan et al., 2020). These findings imply that p53/SLC7A11 pathways play a vital role in sensitizing synovial fibroblasts to ferroptotic cell death.

In this study, the effect of ferroptosis in a RA model and the underlying mechanism of bioactive peptide G1dP3 in inhibiting MH7A cell activation induced by TNF-α were elucidated for the first time. Furthermore, p53/SLC7A11 axis was identified to exert a crucial effect on G1dP3-regulated ferroptosis in MH7A cells.

## Materials and Methods

### Drugs and Cell Death Inhibitors

Ferrostatin-1 was purchased from Selleckchem Company (Shanghai, China). DFO and z-VAD-fmk were supplied by Sigma-Aldrich (MO, United States). The peptides were obtained from Science Peptide Company (Shanghai, China) after purification.

### Cell Culture

MH7A cells (a human RA synovial fibroblast cell line) were obtained from the American Type Culture Collection (VA, United States) and cultured in RPMI 1640 medium (Hyclone, UT, United States) containing 1% penicillin/streptomycin (Invitrogen, CA, United States) and 10% FBS (Gibco, MA, United States) at 37°C and 5% CO_2_. The cells were then induced with 50 ng/ml TNF-α.

### Cell Transfection

The human siRNA-Caspase3 (sc-29237), siRNA-p53 (sc-29435) and negative controls were supplied by Santa Cruz Biotechnology (Shanghai) Co., Ltd. Cell transfection was conducted with Lipofectamine 3,000 (Invitrogen) for 4 h by following the reagent’s protocol. Subsequently, the medium was replenished with a 10% FBS-containing RPMI 1640 medium. After 24 h, the cells were collected. Subsequent western blot was performed to analyze transfection efficiency (Supplementary Figure SA,B).

### Peptide Synthesis

The galectin-1 derived peptide fragment 3 (G1dP3, ADGDFKIK sequences, 95% purity) and the scrambled peptide (ScP, DAGIDKFK sequences, 95% purity) were synthesized with GRKKRRQRRRPPQ sequences derived from the cell-penetrating peptide (CPP) HIV-1 Tat (48–60) (Hu et al., 2020). The peptides (10 mM) were prepared by dissolving in water and kept in a refrigerator at −80°C. G1dP3 and ScP at a concentration of 20 μM were employed for further analysis.

### Cell Viability Assay

Briefly, MH7A cells (5,000 cells/well) were grown in a 96-well plate, and then exposed to the indicated treatment. Cell viability was conducted with Cell Counting Kit-8 (Dojindo Technologies, Kyushu, Japan) by following the kit’s protocol. Subsequently, the absorbance of each well was detected at 450 nm.

### Real-Time Quantitative Polymerase Chain Reaction (RT-qPCR)

Total RNA was isolated with TRIzol reagent (Invitrogen). cDNA synthesis was conducted using a PrimeScript RT Reagent kit (Vazyme Biotech, Nanjing, China) by following the manufacturer’s instructions. RT-qPCR was performed on a 7,900 Real-time PCR system (Applied Biosystems, CA, United States) using the ChamQ Universal SYBR qPCR Master Mix (Vazyme). GAPDH was selected as a housekeeping gene. The relative expression levels were calculated using the 2−ΔΔCT method. The following primer pairs were used:

P53 forward primer: 5′-TTC​CCT​GGA​TTG​GCC​AGA​CT-3′, reverse primer: 5′-ACC​ATC​GCT​ATC​TGA​GCA​GC-3'.

GAPDH forward primer: 5′-AGA​AGG​CTG​GGG​CTC​ATT​TG-3′, reverse primer: 5′-AGG​GGC​CAT​CCA​CAG​TCT​TC-3'.

SLC7A11 forward primer: 5′-TGC​CCA​GAT​ATG​CAT​CGT​CC-3′, reverse primer: 5′-TCT​TCT​TCT​GGT​ACA​ACT​TCC​AGT-3'.

GAPDH forward primer: 5′-CAG​CCT​CAA​GAT​CAT​CAG​CAA​T-3′, reverse primer: 5′-AGT​CCT​TCA​CGA​TAC​CAA​AGT-3'.

### Western Blot Analysis

The protein expression levels were determined by Western blotting after normalization to GAPDH. Briefly, the protein specimens were incubated overnight at 4°C with primary antibodies against BCL-2 (sc-7382, Santa), Bax (sc-7480, Santa), cleaved-Caspase-3 (sc-6053, Santa), Gpx4 (sc-166570,Santa), p53 (sc-126, Santa), SLC7A11 (ab37185, abcam), and GAPDH (sc-365062, Santa).

### Transmission Electron Microscopy (TEM)

TEM analysis was performed according to a previous method (Bao et al., 2021).

### Determination of Labile Iron Pool (LIP)

The cells (1 × 10^6^ cells/ml) were incubated with 0.05 µM calcein-acetoxymethyl ester (AnaSpec) at 37°C for 15 min. After rinsing twice with 0.5 ml PBS, the cells were treated with/without 100 µM deferiprone at 37°C for 1 h. Flow cytometric analysis at 525 nm emission and 488 nm excitation. The levels of LIP were determined by comparing the mean fluorescence intensities between deferiprone treated and untreated groups.

### Detection of ROS and Lipid ROS

The intracellular ROS levels were measured by DCF-DA (2′,7′-dichlorodihydrofluorescein diacetate; Molecular Probes/Invitrogen, OR, United States) by following the manufacturer’s protocols. Briefly, after incubation with 20 μM DCF-DA at 37°C for 30 min, the fluorescence signals of the cells were determined using a FACStar Flow Cytometer (Beckman Coulter). Lipid ROS levels were evaluated using the BODIPY 581/591 C11 (D3861, Invitrogen) by following the manufacturer’s protocols.

### Measurement of Reduced Glutathione (GSH)/Oxidized Glutathione (GSSG) Activity

The ratio of GSH/GSSG was detected using the GSH/GSSG-Glo Assay (Promega, United States). After incubation with 50 μl Oxidized Glutathione Lysis Reagent or Total Glutathione Lysis Reagent for 5 min, the cells were incubated again for 30 min with 50 μl Luciferin Generation Reagent. Then, 100 μl Luciferin Detection Reagent was added, and the luminescence signals were detected using a microplate reader (BioTek).

### Statistical Analysis

Statistical tests were conducted with SPSS v16.0 (SPSS Inc., United States). All data were presented as mean ± standard deviation. Unpaired Student t-test was applied to compare the difference between two groups. One-way ANOVA was employed to compare the differences among multiple groups. *p* < 0.05 was regarded as statistically significant.

## Results

### G1dP3 Inhibits MH7A Cell Viability Independent of Apoptosis

**FIGURE 1:**
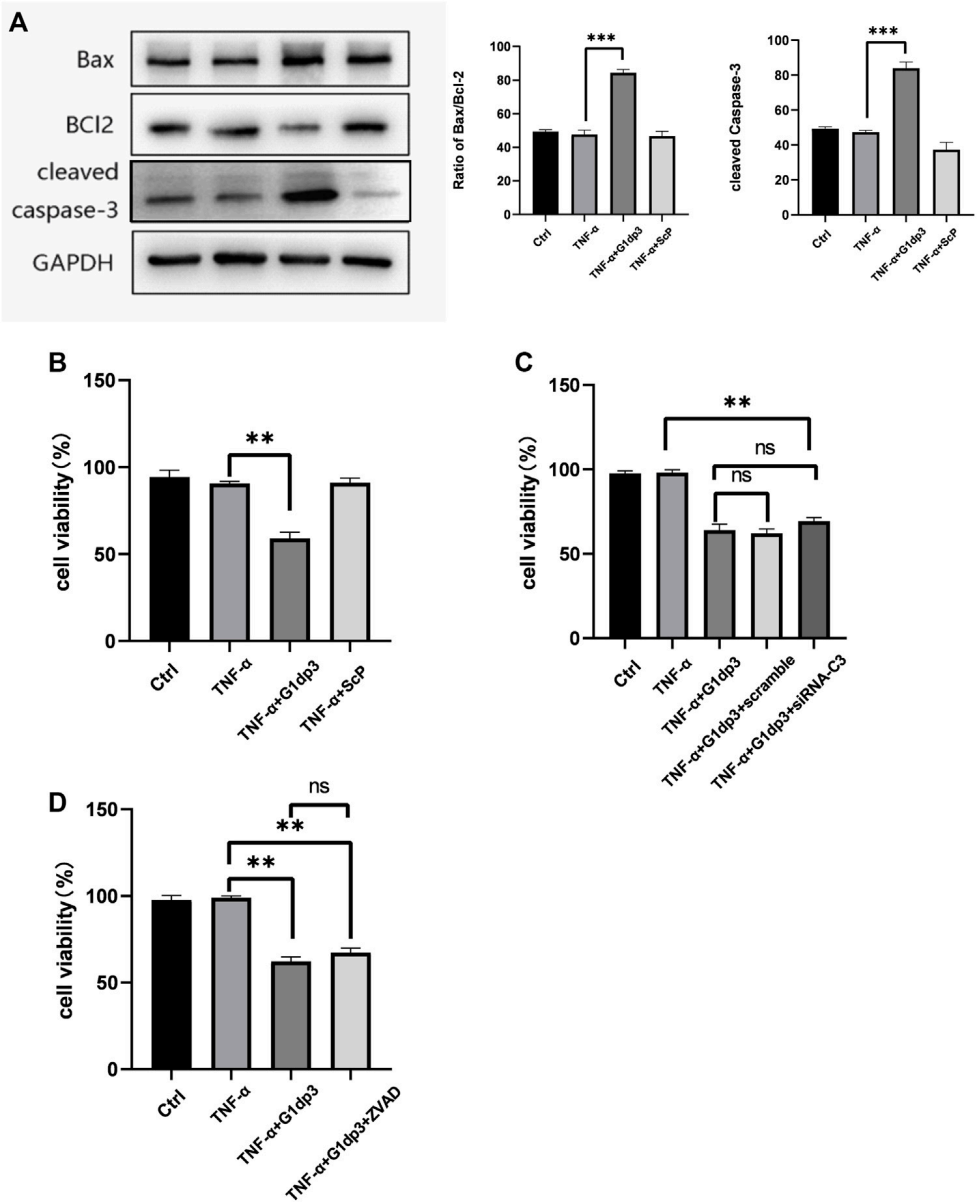
G1dP3 inhibits MH7A cell viability independent of apoptosis. **(A)** The protein levels of Bax, cleaved-caspase-3 and Bcl-2. G1dP3 (20 μM, 6 h) dramatically increased the expression of Bax and cleaved-caspase-3, while decreased that of Bcl-2. **(B)** Cell viability was detected in the indicated MH7A cells. MH7A cell viability was inhibited by G1dP3 (20 μM, 6 h). **(C,D)** The inhibition of G1dP3 in MH7A cell viability could not be reversed by siRNA-Caspase3 (20 μM) or pre-treated z-VAD-fmk (30 μM, 24 h). Graph represents mean ± SD; *p < 0.05, **p < 0.01, ***p < 0.001, ns, not significant. Representative data from one of three individual experiments.

In this study, 50 ng/ml TNF-α was used to establish a RA model using the MH7A cells. Consistent with our previous results, treatment of 20 μM G1dP3 for 6 h significantly up-regulated the expression of Bax and cleaved-caspases-3, while down-regulated that of Bcl-2 in MH7A cells (Figure 1A). Moreover, G1dP3 treatment resulted in a reduction of cell viability compared to the TNF-α model group (Figure 1B). To determine whether G1dP3-suppressed MH7A cell viability was mediated by the apoptotic process, z-VAD-fmk (30 μM) and siRNA-caspase-3 (20 μM) were used to inhibit apoptosis and knockdown the major executors of apoptosis, respectively. Interestingly, the inhibitory effect of G1dP3 on MH7A cell viability could not be reversed by blocking apoptosis (Figures 1C,D). These findings suggest that G1dP3 suppresses MH7A cell viability independent of apoptosis.

### The Inhibitory Effect of G1dP3 on MH7A Cell Viability Is Correlated With Ferroptosis

**FIGURE 2:**
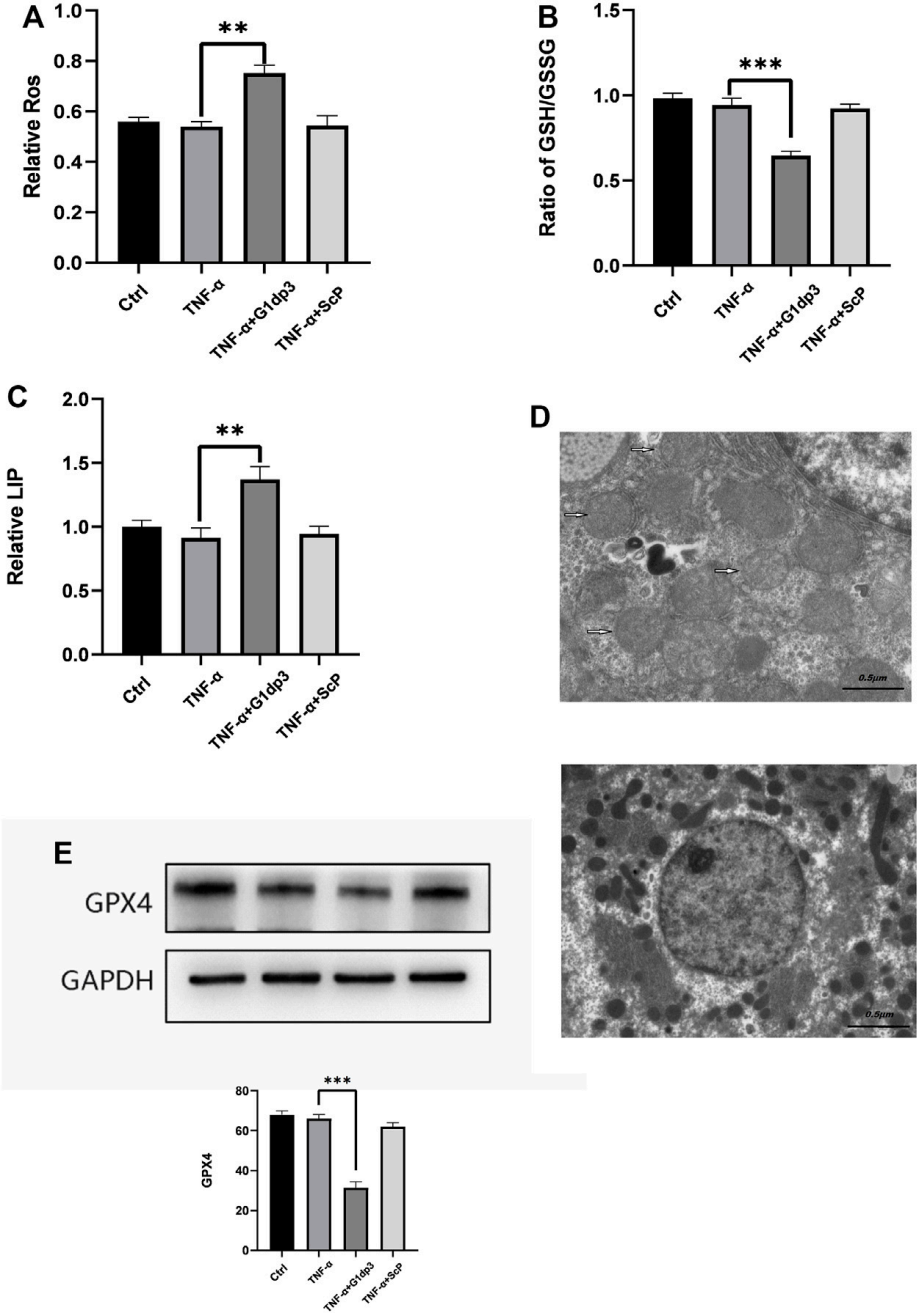
The inhibitory effect of G1dP3 on MH7A cell viability is correlated with ferroptosis **(A)** Relative lipid peroxidation levels in the indicated MH7A cells assessed by BODIPY 581/591 C11. G1dP3 (20 μM, 6 h) could increase the accumulation of lipid ROS. **(B)** Ratio of GSH/GSSG in the indicated MH7A cells. G1dP3 reduced the ratio of GSH/GSSG. **(C)** Relative cellular labile iron pool (LIP). G1dP3 could significantly increase free iron levels in MH7A cells. **(D)** Representative TEM images illustrating that G1dP3 alters mitochondrial morphology in MH7A cells. Scale bars are displayed in each image. **(E)** The protein expression of Gpx4. Graph represents mean ± SD; *p < 0.05, **p < 0.01, ***p < 0.001, ns, not significant. Representative data from one of three individual experiments.

Different from necroptosis and apoptosis, nerroptosis is a newly discovered type of regulated cell death (RCD), which is independent of receptor-interacting protein 1 (RIPK1) kinase activity and caspase activity. In the present study, we hypothesize that G1dP3 plays a vital role in regulating MH7A cell death via ferroptosis induction. We firstly examined the levels of ROS in TNF-α-induced MH7A cells following G1dP3 treatment. The results showed that G1dP3 could increase the accumulation of lipid ROS assessed by BODIPY 581/591 C11 (Figure 2A). Moreover, G1dP3 markedly reduced the ratio of GSH/GSSG (Figure 2B). In addition, G1dP3 could significantly increase free iron levels by measuring the cellular labile iron pool (LIP) (Figure 2C). TEM was utilized to observe the cell death mode. Compared to the TNF-α group, G1dP3-treated MH7A cells showed smaller, ruptured mitochondria with increased membrane density and vanished mitochondria cristae, thereby conferring morphological changes in ferroptosis cells (Figure 2D). As we expected, Western blot analysis revealed that the protein expression of Gpx4 (a ferroptosis marker) was also decreased after G1dP3 treatment (Figure 2E). Collectively, these results indicate that G1dP3 regulates ferroptosis in MH7A cells.

### Inhibition of Ferroptosis Alleviates G1dP3-Induced Suppression of MH7A Cell Viability

**FIGURE 3:**
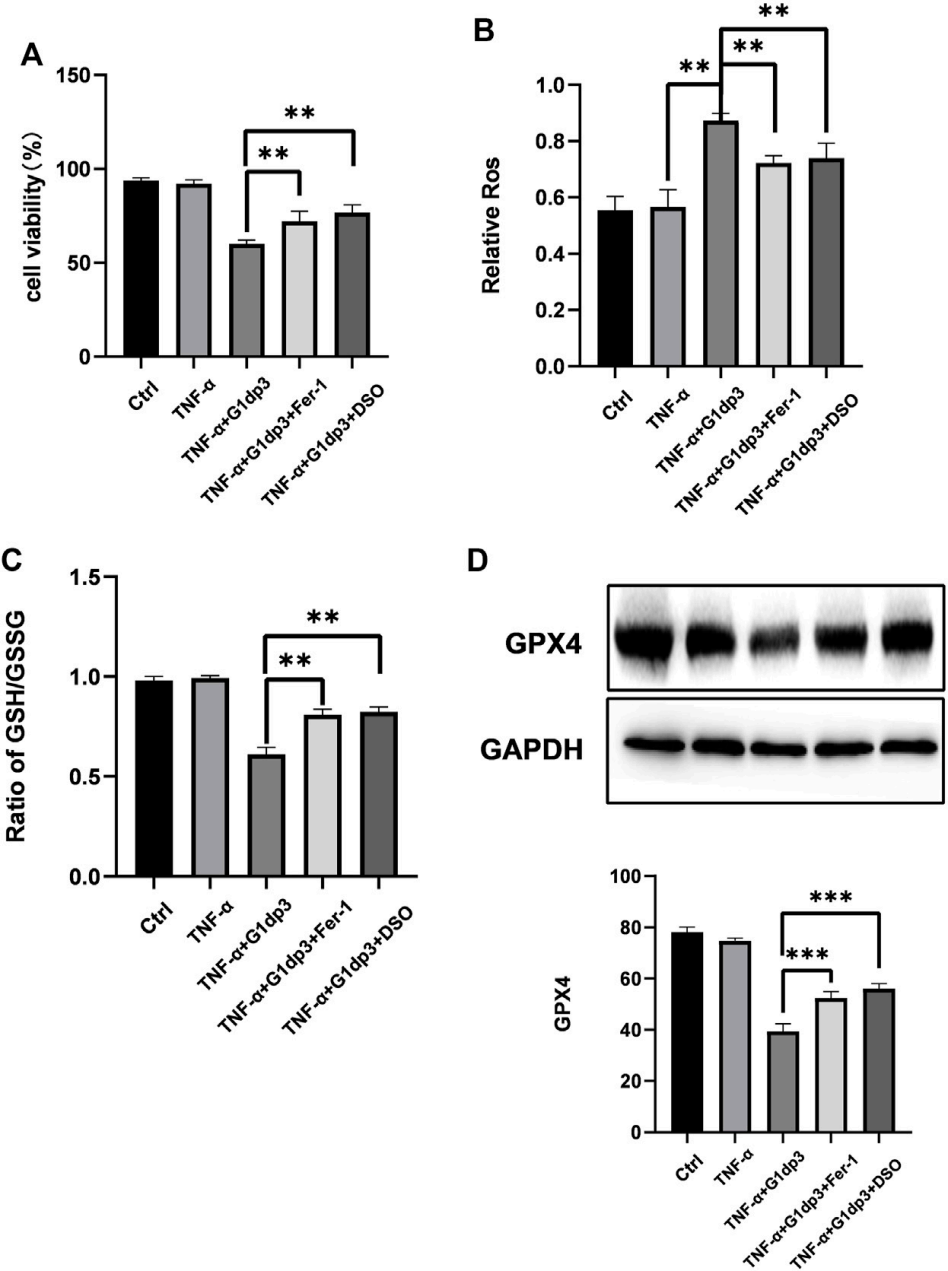
Inhibition of ferroptosis alleviates G1dP3-induced suppression of MH7A cell viability MH7A cells were pretreated with the ferroptosis-specific inhibitor Fer-1 (2 μM), iron chelator DFO (80 μM) for 24 h, and then coincubated with G1dP3. **(A)** Cell viability was detected in the indicated MH7A cells. **(B)** Relative lipid peroxidation levels in the indicated MH7A cells. **(C)** Ratio of GSH/GSSG in the indicated MH7A cells. **(D)** Protein levels of Gpx4 in the indicated treatment. Graph represents mean ± SD; *p < 0.05, **p < 0.01, ***p < 0.001, ns, not significant. Representative data from one of three individual experiments.

To further investigate whether ferroptosis was involved in G1dP3-mediated MH7A cell viability suppression, we consequently treated MH7A cells with ferroptosis specific inhibitor, ferrostatin-1 (Fer-1, 2 μM) and iron chelators, deferoxamine (DFO, 80 μM) for 24 h to block the initiation of ferroptosis. As shown in Figure 3A, G1dP3-induced suppression of MH7A cell viability was remarkably restored by Fer-1 and DFO, along with lipid ROS depletion and GSH generation (Figures 3A–C). Likewise, Western blot results indicated that both Fer-1 and DFO could reverse G1dP3-induced Gpx4 down-regulation (Figure 3D). Taken together, these findings imply that the suppression of MH7A cell activity is associated with G1dP3-induced ferroptosis.

### G1dP3 Regulates Ferroptosis Through p53/SLC7A11 Axis

**FIGURE 4:**
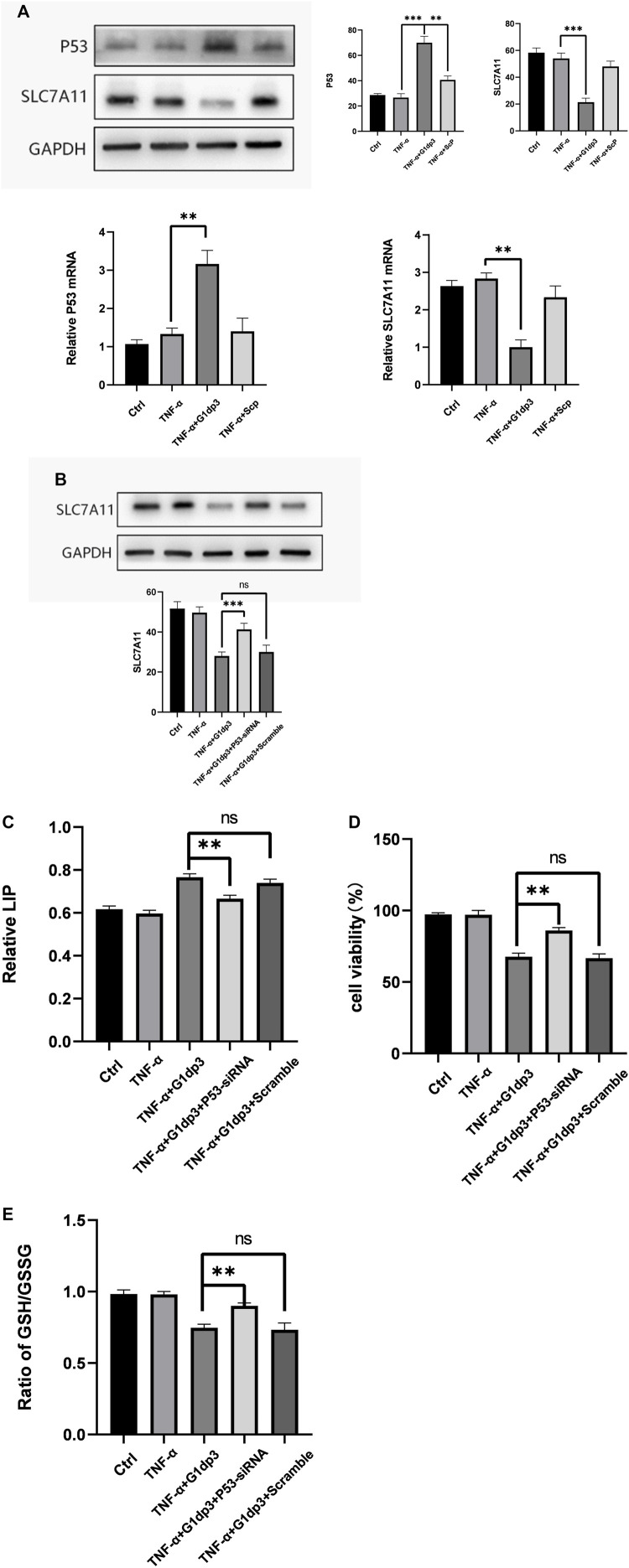
(Continued).

P53 can serve as an essential factor for regulating ferroptosis in response to different types of damage. SLC7A11 is a target gene of p53. xCT, the predominant transporter of cystine, is encoded by the SLC7A11 gene. In the cells, cystine is reduced to cysteine, which is a key molecule for GSH synthesis. Depletion of GSH by xCT inhibition could serve as a crucial pathway for the development of ferroptosis. To determine whether p53/SLC7A11 axis can govern ferroptotic activity in G1dP3-treated MH7A cells, the expression levels of p53 and SLC7A11 were detected. As shown in Figure 4A, G1dP3 increased p53 but decreased SLC7A11 expression at both mRNA and protein levels. To confirm the role of p53 in G1dP3-induced ferroptosis, p53 was knocked down through specific small interfering RNA. It was found that siRNA-p53 could significantly increase SLC7A11 expression (Figure 4B). Furthermore, knockdown of p53 markedly decreased G1dP3-induced free iron levels (Figure 4C). On the contrary, siRNA-p53 significantly enhanced cell viability and GSH/GSSG ratio in G1dP3-treated MH7A cells (Figures 4D,E). These data reveal that blockade of p53 activation markedly abrogates the ferroptotic effects of G1dP3, and p53/SLC7A11 axis plays a pivotal role in regulating G1dP3-induced ferroptosis in MH7A cells.

## Discussion

RA is a complex autoimmune disorder characterized by chronic synovitis and gradually leads to the destruction of articular cartilage, even the corruption of bone and the impairment of joints. Although the etiology of RA remains unclear, recent evidence has suggested that activation of RA synovial fibroblasts, a complex network of cell regulation, plays a vital role in the pathogenesis of RA (Pap et al., 2000; Turner and Filer, 2015). Thus, inhibiting RA synovial fibroblast proliferation and blocking its effects can significantly reduce RA symptoms and prevent bone destruction (Panipinto et al., 2021).

As a new branch of proteomics, peptidomics can be used to analyze endogenous protein fragments in body fluids and tissues (Smolen et al., 2010). Over the past decades, biologically active peptides have gained considerable interest as a therapeutic agent, since their are highly selective, efficacious, relatively safe and well-tolerated (Fosgerau and Hoffmann, 2015; Zorko et al., 2022). Although bioactive peptides are responsible for nearly all physiological processes such as tumor formation, immune regulation and cell differentiation, the potential role of endogenous peptides in the pathophysiology of RA synovial fibroblasts remains unclear. At present, there is a lack of peptidomics studies on RA synovial tissues. Our previous study showed that G1dP3 had potent anti-proliferation and anti-inflammation effects on MH7A synovial cells induced by TNF-α, indicating that it has great potential for the treatment of RA. In this study, the underlying mechanisms of G1dP3 in inhibiting MH7A cell viability was elucidated. Given that the peptides are unable to cross cell membranes and exert their functions, G1dP3 with a CPP attached to its N-terminal was chemically synthesized for further research (8). G1dP3 treatment remarkably up-regulated the expression of Bax and cleaved-caspase-3, while down-regulated that of Bcl-2 in MH7A cells induced by TNF-α. Caspase-3 is the major executor of apoptosis (Lim et al., 2010), and the balance of protein Bcl-2 and Bax plays an essential role in regulating mitochondrial-dependent apoptotic pathways (Tian et al., 2016), Therefore, we primarily assume that apoptotic process is the potential mechanism of G1dP3 in reducing MH7A cell viability. To validate this conjecture, z-VAD-fmk, an inhibitor of apoptosis, was employed. Moreover, the expression of caspase-3 was knocked down with siRNA-caspase3 to prevent apoptosis execution. However, blocking apoptosis induction did not attenuate the anti-proliferation effect of G1dP3 on MH7A cells, suggesting that G1dP3 could inhibit MH7A cell viability independent of apoptosis.

Ferroptosis is a newly recognized form of RCD discovered by Brent R. Stockwell’s lab in 2012. Distinct from other types of RCD (e.g., autophagy, necrosis and apoptosis) at morphological, genetical and mechanistic levels, ferroptosis is defined as an iron-dependent form of non-apoptotic cell death (Dixon et al., 2012; Jiang et al., 2021). Mechanistically, ferroptosis arises from dysregulated lipid peroxidation or iron metabolism, along with GSH depletion and Gpx4 inactivation (Dixon et al., 2012; Jiang et al., 2021). Neither suppression of apoptosis, necrosis nor autophagy by small molecules can reverse ferroptosis. Meanwhile, cellular morphological manifestations in ferroptosis cells are also different from normal features. More importantly, rupture of the mitochondrial outer membrane and deposition of copious lipid droplets in the cytoplasm are dispensable for ferroptosis (Jiang et al., 2015b). A growing body of research has pointed out the function of ferroptosis in degenerative diseases (Stockwell et al., 2017). For instance, inhibition of ferroptosis could alleviate traumatic and hemorrhagic brain injury and other neurodegeneration syndromes (Stockwell et al., 2017; Bao et al., 2021). Besides, ferroptosis may be a potential therapeutic target for ameliorating hemochromatosis-related tissue damage and liver fibrosis (Wang et al., 2017, 2019). It seems that degenerative diseases are induced by disorders mainly in repairing peroxidized lipids, which subsequently lead to cell death (Viswanathan et al., 2017; Tarangelo et al., 2018). Thus, ferroptosis might serve as a suppressor or inducer in degenerative disorders by removing cells lacking access to seize crucial nutrients, including chondrocyte death and bone erosion in RA (Maiorino et al., 2018). To our surprise, transmission electron microscopy showed smaller, ruptured mitochondria with increased membrane density and vanished mitochondria cristae, which conferred morphological changes in ferroptosis cells. These findings implicated that G1dP3-mediated MH7A cell viability suppression was associated with cellular ferroptosis. As expected, the enhanced lipid peroxidation, elevated iron levels, depleted GSH levels and reduced Gpx4 activity were also observed in G1dP3-mediated MH7A cells. Furthermore, the ferroptosis specific inhibitor Fer-1 and iron chelators DFO alleviated G1dP3-induced decrease of MH7A cell viability. Taken together, these data support our hypothesis that the suppression of MH7A cell viability is associated with G1dP3-regulated ferroptosis. To our knowledge, this is the first time report of ferroptosis in RA synovial fibroblasts.

**FIGURE 5:**
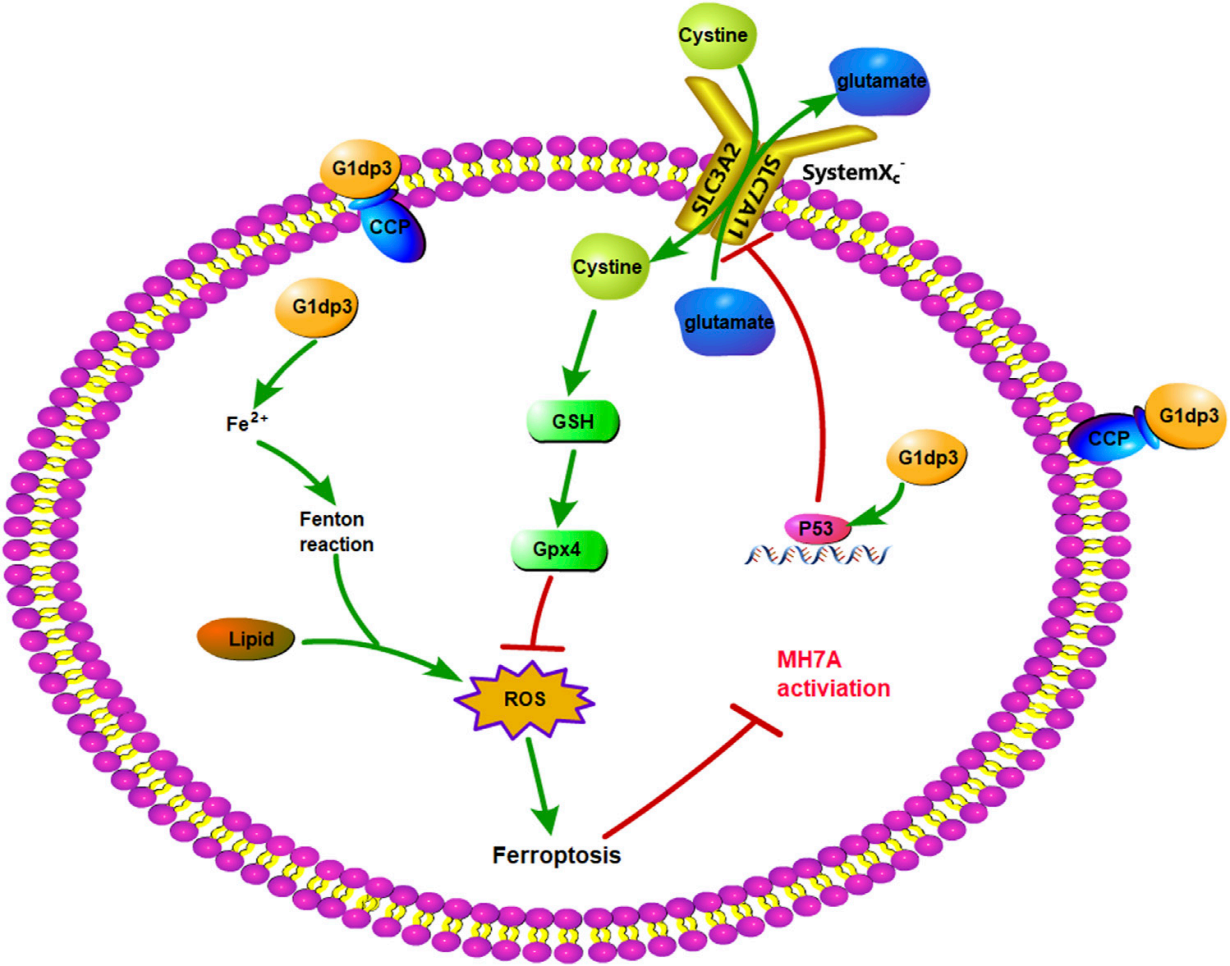
p53/SLC7A11 pathways play an essential role in G1dP3-regulated MH7A ferroptosis. G1dP3 treatment induced p53 overexpression, SLC7A11 inactivation, GSH depletion, Gpx4 deficiency, ROS production, ferroptosis activation, and ultimately suppressed MH7A cell activation.

Commonly referred to as the “guardian of the genome”, p53 has been considered a powerful tumour suppressor through the induction of apoptosis, senescence and cell cycle arrest (Levine and Oren, 2009; Levine, 2020). However, the single function of p53 responsible for its tumor suppressive effects remains to be identified (Kaiser and Attardi, 2018). Recently, Gu et al. revealed that p53 suppressed tumour growth through its metabolism-regulatory ability, instead of its DNA-damage response (Liu and Gu, 2022). p53 can regulate a cascade of metabolic pathways including amino acids, lipid, ROS and iron metabolism, which is tightly bound to ferroptosis (Stockwell et al., 2020; Liu and Gu, 2021). Furthermore, p53 was proposed to positively regulate ferroptosis by downregulating SLC7A11 expression to induce the levels of ROS (Jiang et al., 2015b; Wang et al., 2019). SLC7A11 is an active constituent of the system X_c_^−^, which is a disulfide-bonded heterodimer generated via SLC3A2 and SLC7A11 (Sato et al., 1999). The system X_c_^−^ has antioxidant properties by supplying cysteine for GSH synthesis and maintaining redox balance across the plasma membranes (Conrad and Sato, 2012). As a consequence, SLC7A11 is mainly involved in the negative modulation of ferroptosis (Jiang et al., 2015a; Wang et al., 2016). Additionally, p53^K101R^ mutant has lost its ability to induce SLC7A11 expression (Liu et al., 2019), indicating that p53/SLC7A11 signaling may govern ferroptotic activity in MH7A cells. Our findings demonstrated that G1dP3 increased the expression and nuclear transportation of p53, accompanied by the downregulated expression of SLC7A11 in both mRNA and protein levels. Knockdown of p53 abrogated the ferroptotic effects of G1dP3 on MH7A cells by decreasing free iron levels, promoting GSH synthesis, and in turn enhancing MH7A cell viability. Overall, these findings revealed that G1dP3 mediated ferroptosis in TNF-α-induced MH7A cells via a p53/SLC7A11 axis-dependent mechanism (Figure 5).

Nevertheless, some limitations exist in our study. Firstly, although the inhibitory effect of G1dP3 on MH7A cell viability is independent of apoptosis, the proapoptotic familiy gene members were indeed upregulated and the executor of apoptosis Caspase-3 cleaved after G1dP3 treatment. This indicates that G1dP3 induces ferroptosis, accompanying with apoptotic regulation. Hence, other metabolic mechanisms of RA synovial fibroblasts should be explored in further studies. What’s more, as a classic apoptosis-promoting gene, the increased expression and nuclear transportation of p53 following the treatment of G1dP3 may account for the upregulation of apoptotic genes (Figure 1). Our findings also confirmed that blockade of p53 activation markedly abrogated the ferroptotic effects of G1dP3 by promoting the expression of SLC7A11. However, the expression levels of apoptosis-related genes after p53 knockdown have not been detected. Moreover, ferroptosis proceeds even in the absence of key apoptotic effectors, (e.g., caspases and BAX/BAK), which has been regarded as an non-apoptotic form of RCD driven by lipid peroxidation and iron deposition. It has been previously suggested that the inhibition of caspase or deletion of BAX/BAK should be examined to confirm the effect of apoptosis or ferroptosis, as both apoptosis and ferroptosis may simultaneously occur during the pathological process of cell death (Stockwell et al., 2017). Ferroptotic cells are characterized by smaller, ruptured mitochondria with increased membrane density and vanished mitochondria cristae, which may be regulated by the proapoptotic BCL2 family members such as BCL2-binding component 3 (BBC3, also known as PUMA) (Hong et al., 2017) and BH3-interacting domain death agonist (BID) (Neitemeier et al., 2017), indicating the possibilities of interaction between ferroptosis and apoptosis in cell metabolism. Furthermore, necroptotic pathways may be revealed by the suppression of apoptosis via caspase inhibition, and similar backup systems will come into action when other RCD modalities are suppressed (Tang et al., 2019). Thus, more research is needed to explore the interplay between ferroptosis and apoptosis and identify the unique molecular effectors of ferroptosis following apoptosis suppression.

## Conclusion

Our data demonstrated for the first time that the bioactive peptide G1dP3 could sensitize RA synovial fibroblasts to ferroptotic cell death via a p53/SLC7A11 axis-dependent mechanism, thereby providing novel opportunities and future directions for RA treatment. However, further studies are required to validate our findings in terms of RA prevention through an *in vivo* model.

## Data Availability

The original contributions presented in the study are included in the article/Supplementary Material, further inquiries can be directed to the corresponding author.

## Supplementary Material

Supplementary Figure SA Western blot analysis of protein expression of p53. MH7A were pretreated with p53-siRNA or siRNA. **p<0.01.

Supplementary Figure SB Western blot analysis of protein expression of Caspase3. MH7A were pretreated with Caspase3-siRNA or siRNA. **p<0.01.
