# Supplementary figures and images for: p53: A Regulator of Ferroptosis Induced by Galectin-1 Derived Peptide 3 in MH7A Cells
Source: Front Genet. 2022 Jul 4;13:920273. doi: 10.3389/fgene.2022.920273 (PMC9289366; doi:10.3389/fgene.2022.920273)

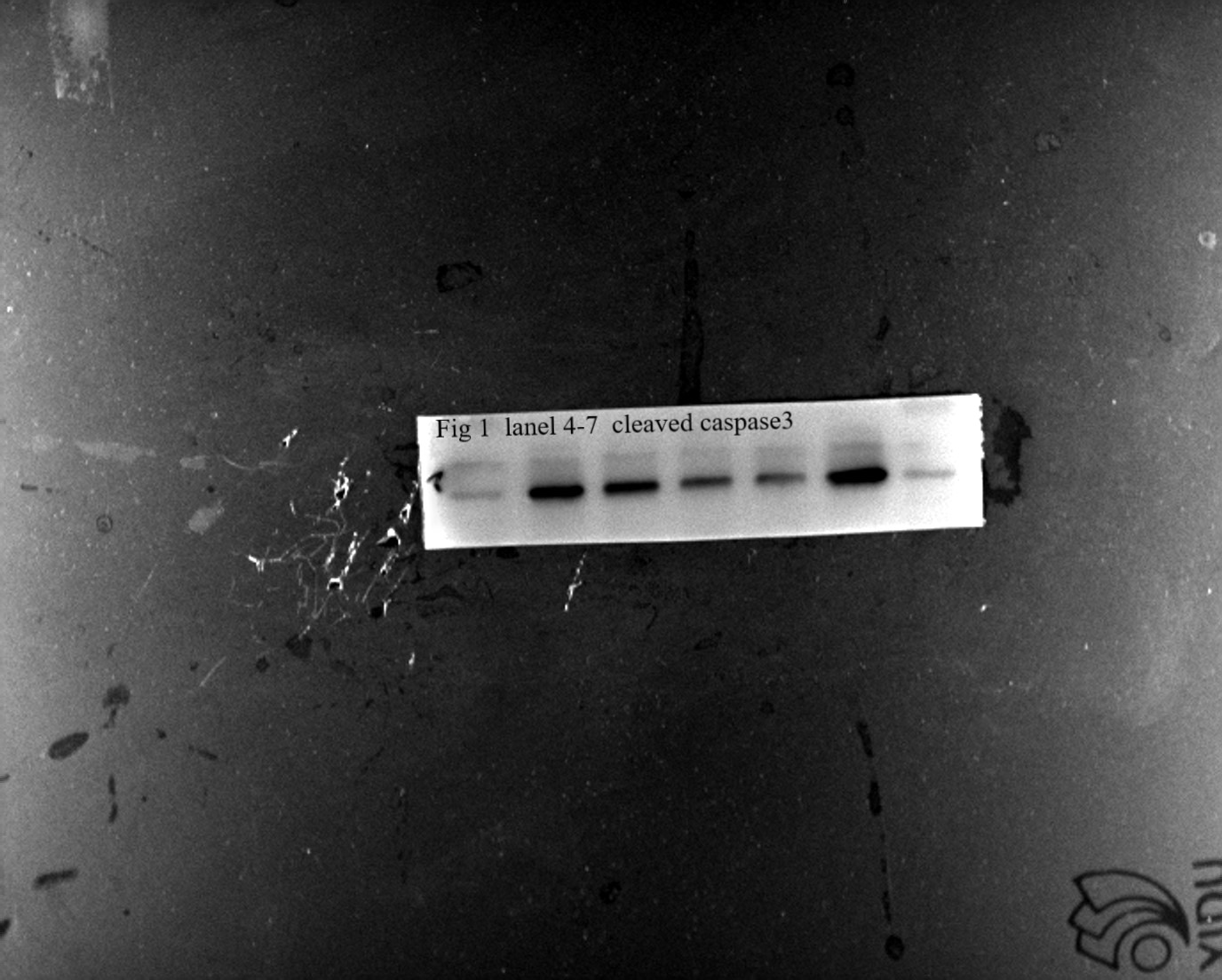

Supplement: Supplementary file 1 [file Image3.JPEG]

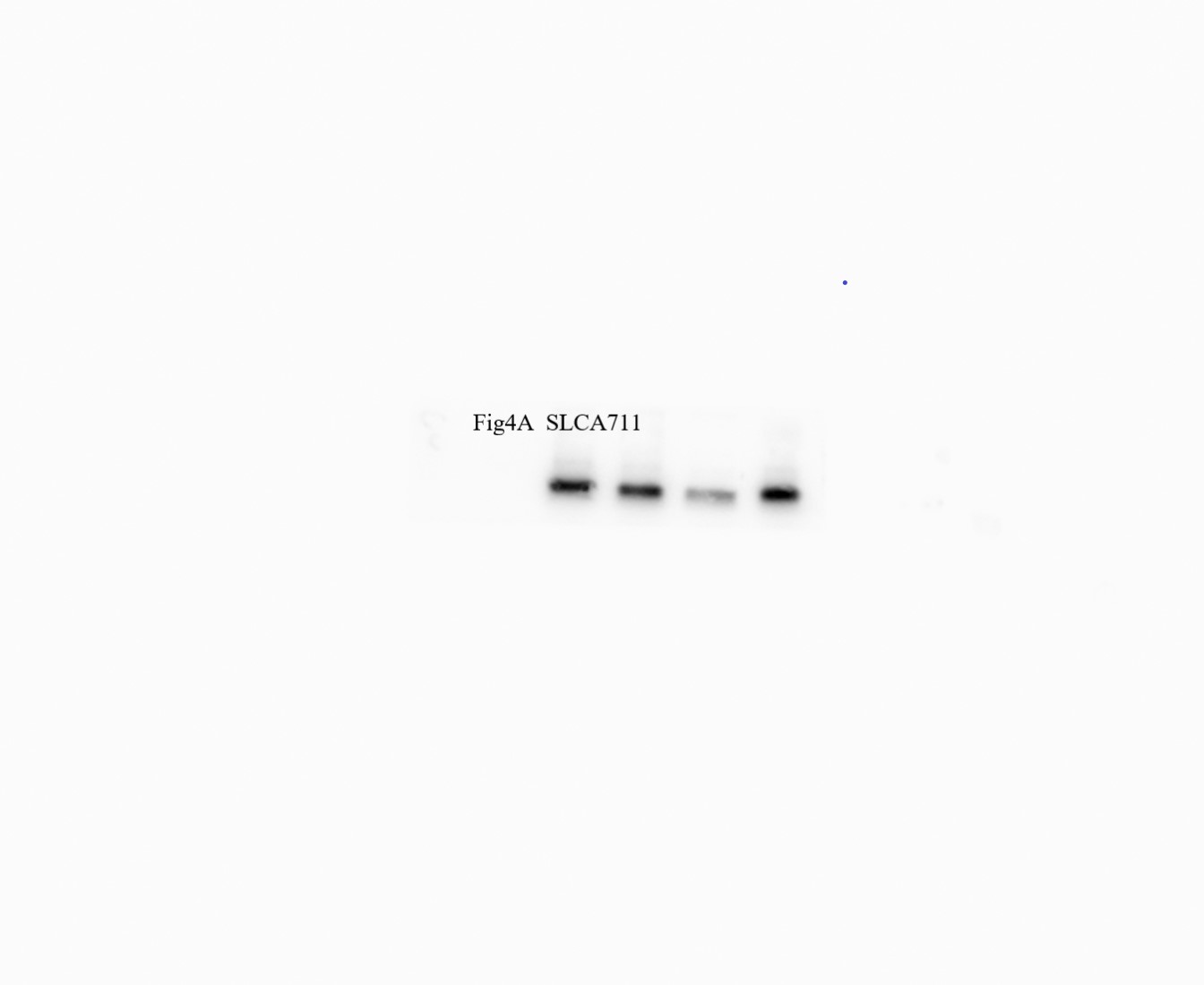

Supplement: Supplementary file 2 [file Image9.JPEG]

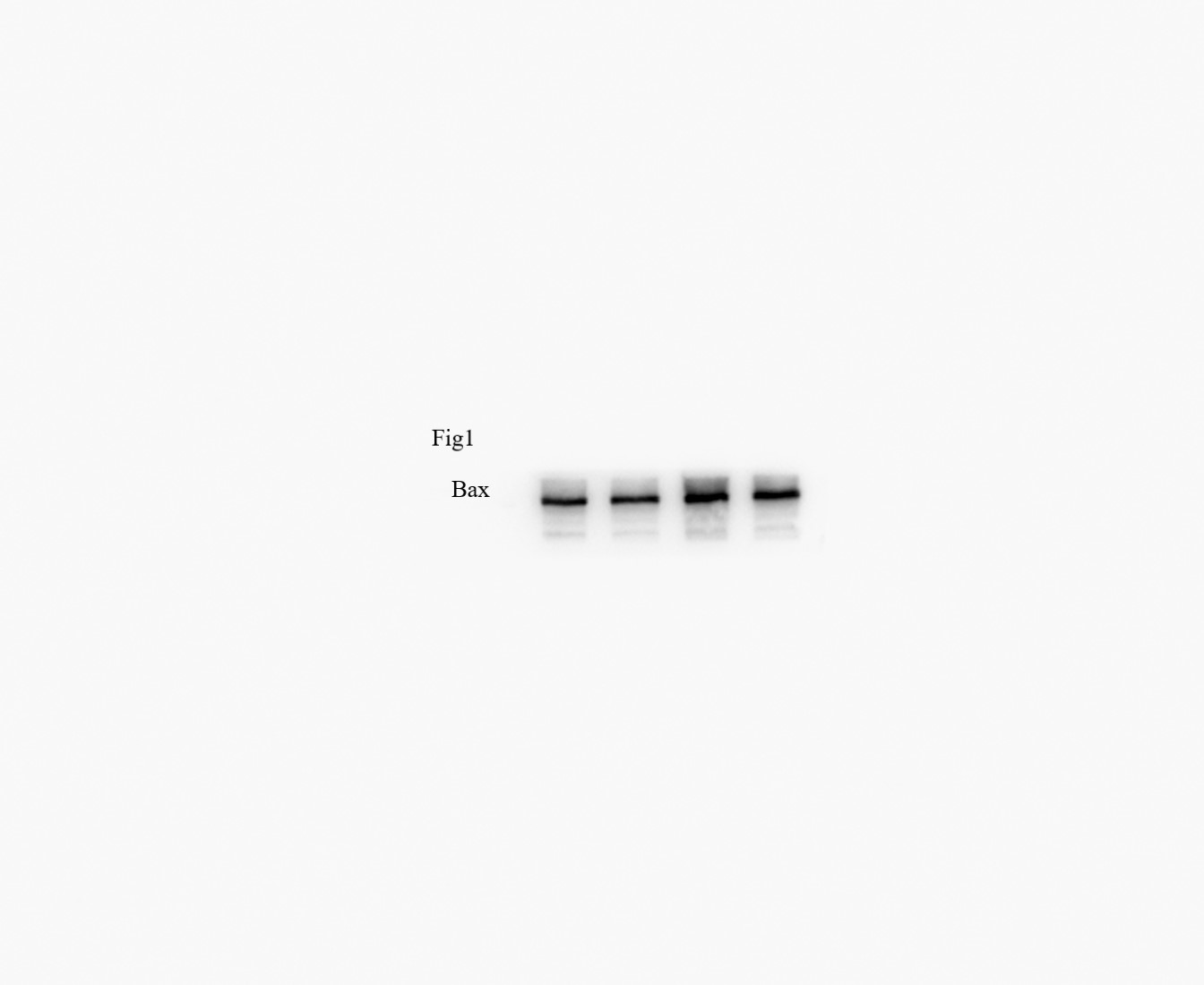

Supplement: Supplementary file 3 [file Image4.JPEG]

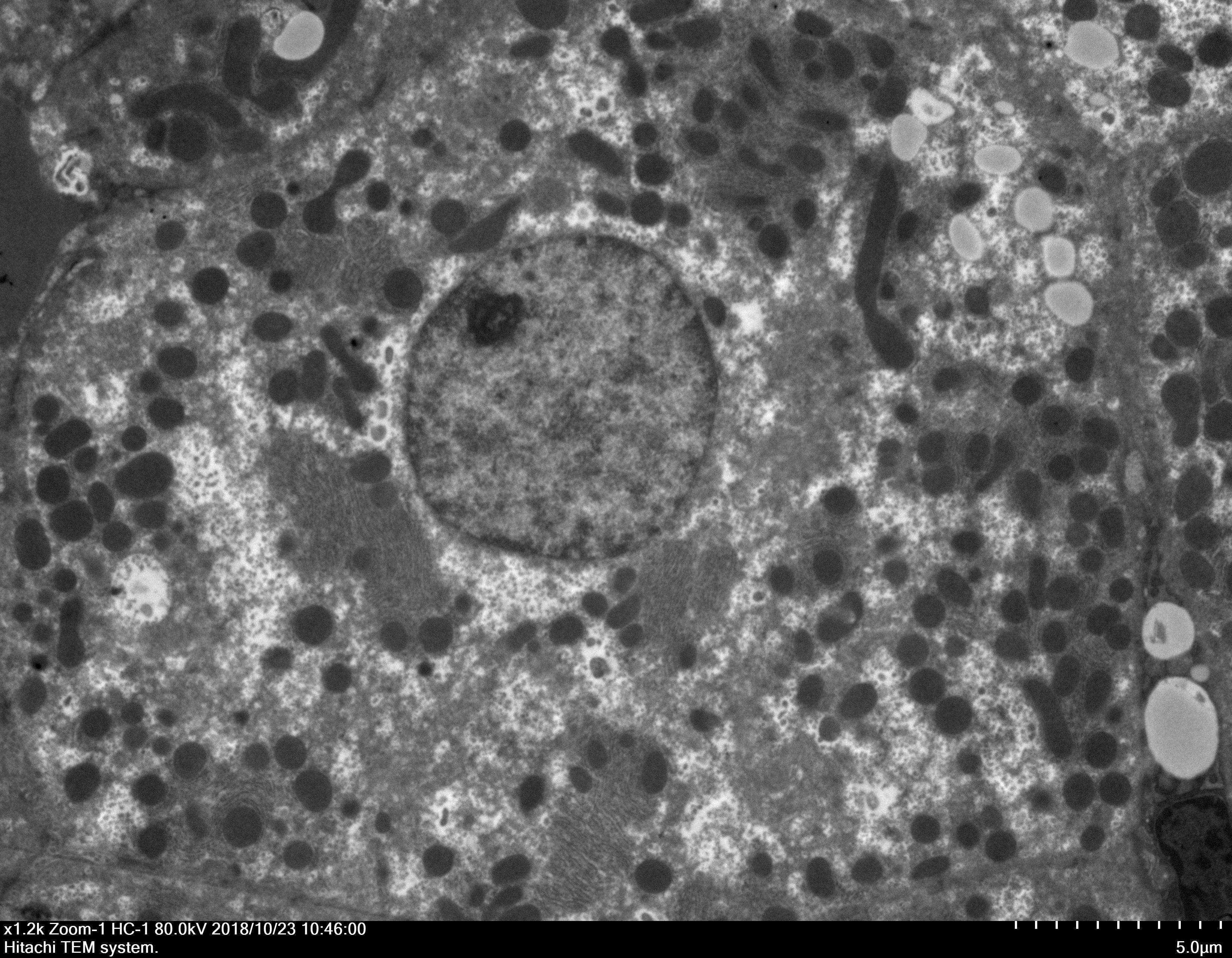

Supplement: Supplementary file 5 [file Image2.TIF]

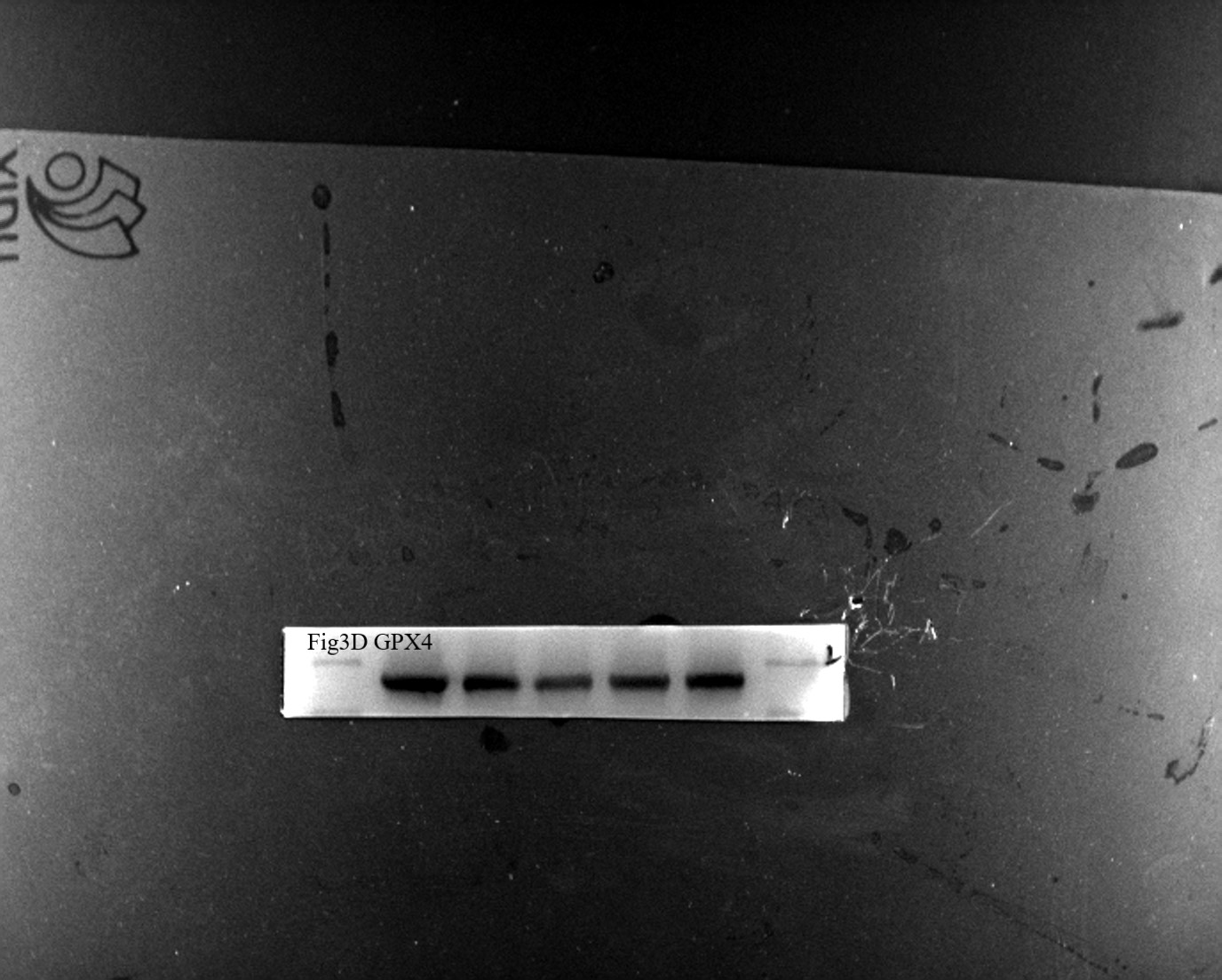

Supplement: Supplementary file 6 [file Image7.JPEG]

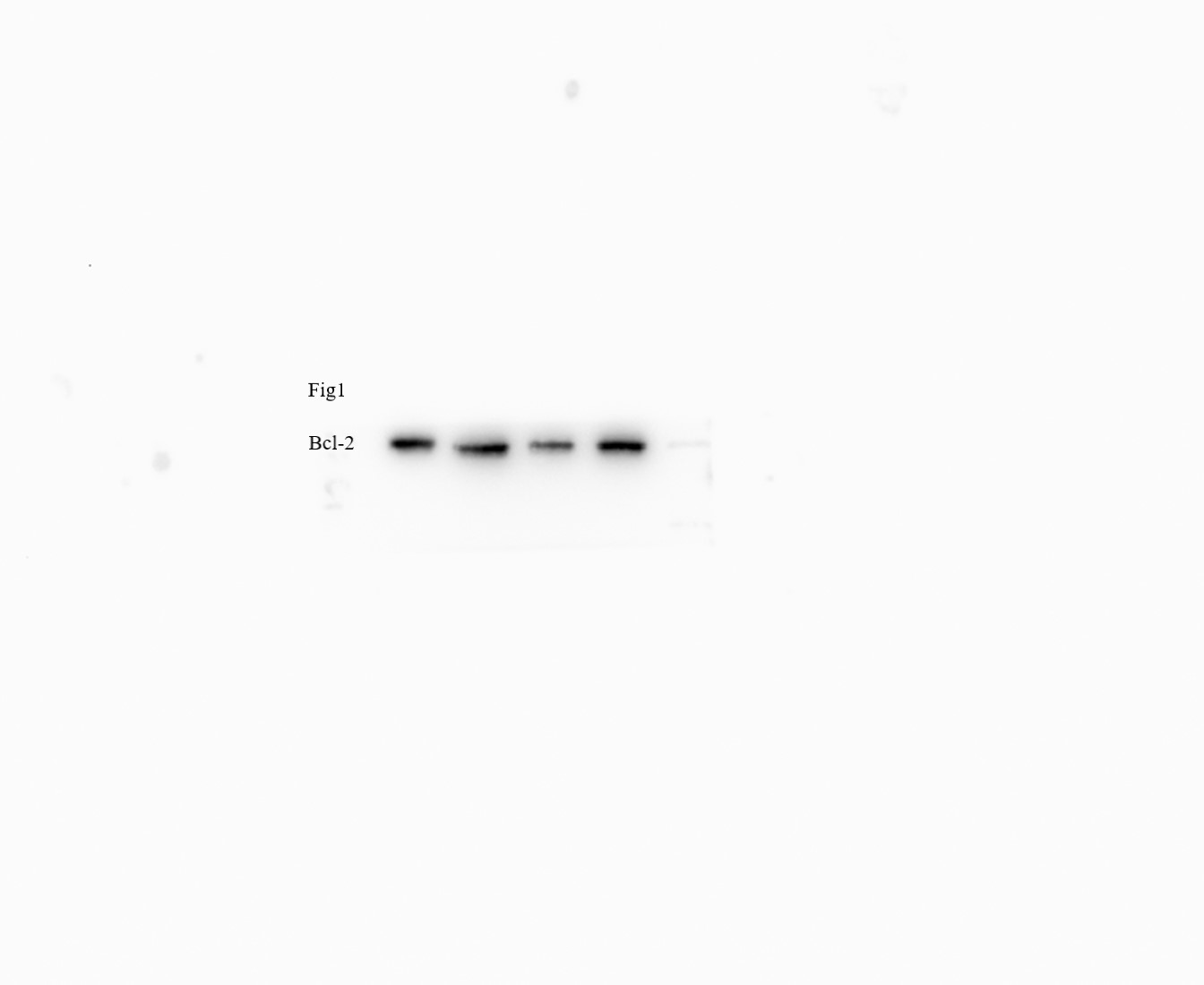

Supplement: Supplementary file 7 [file Image5.JPEG]

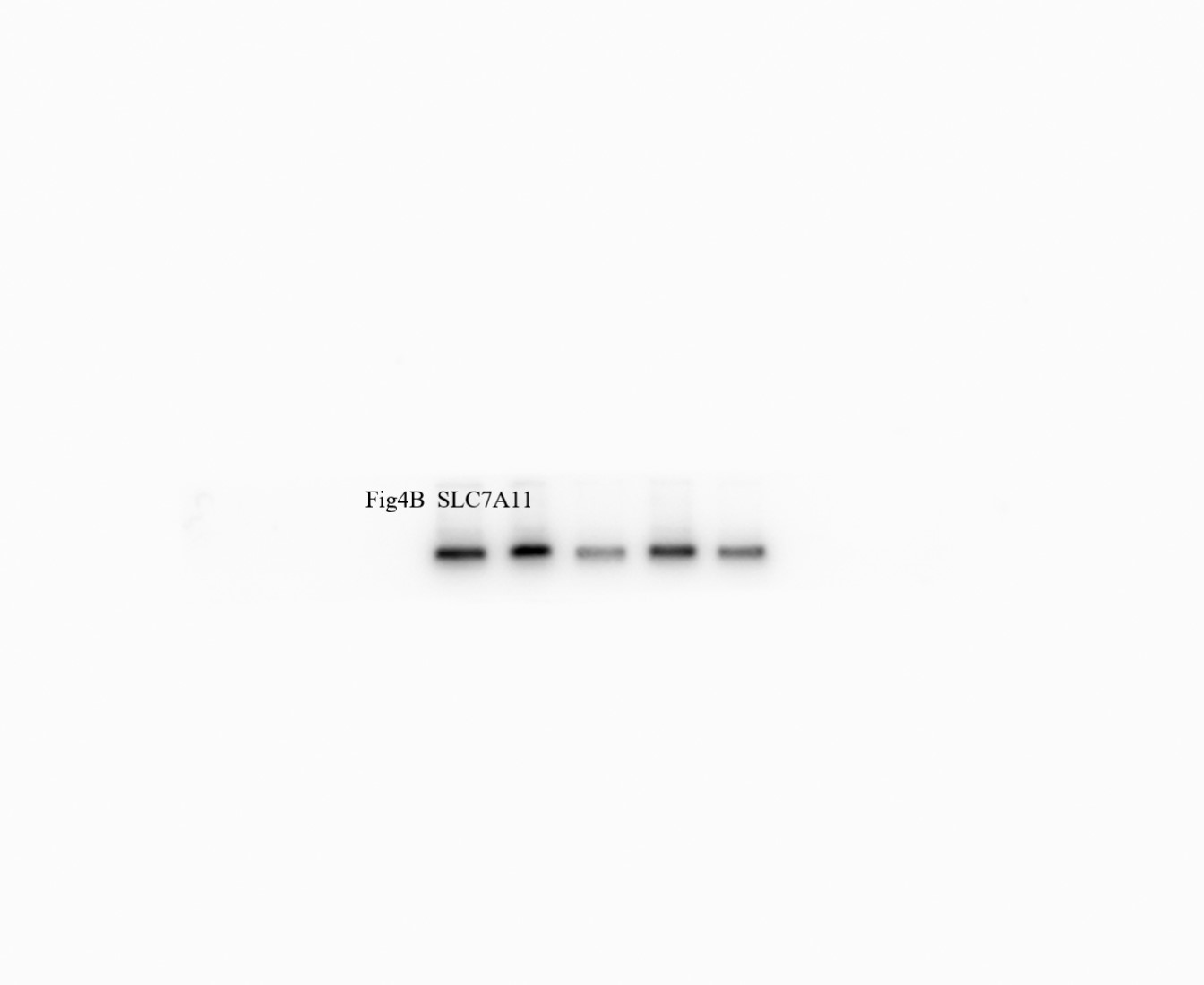

Supplement: Supplementary file 8 [file Image10.JPEG]

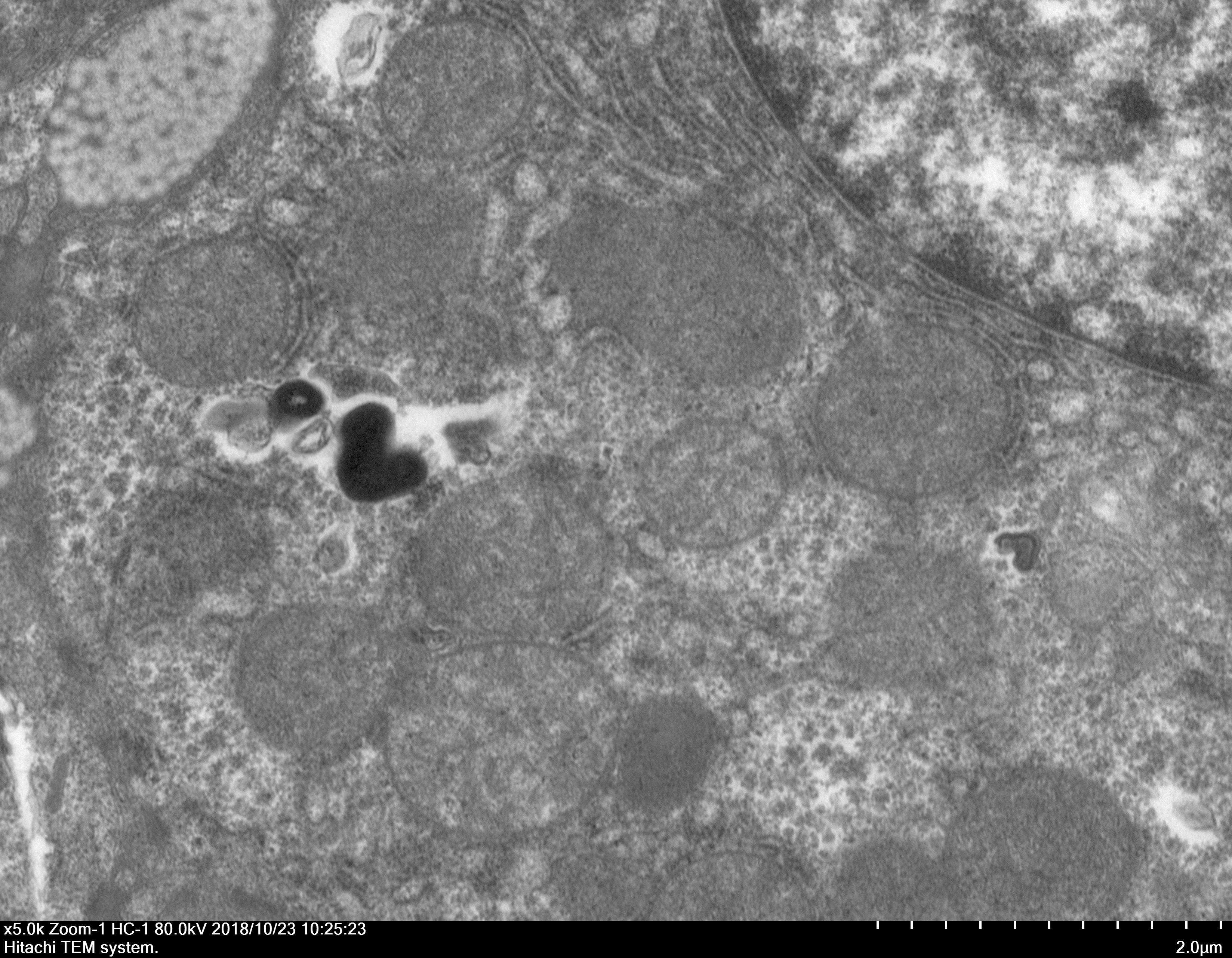

Supplement: Supplementary file 9 [file Image1.TIF]

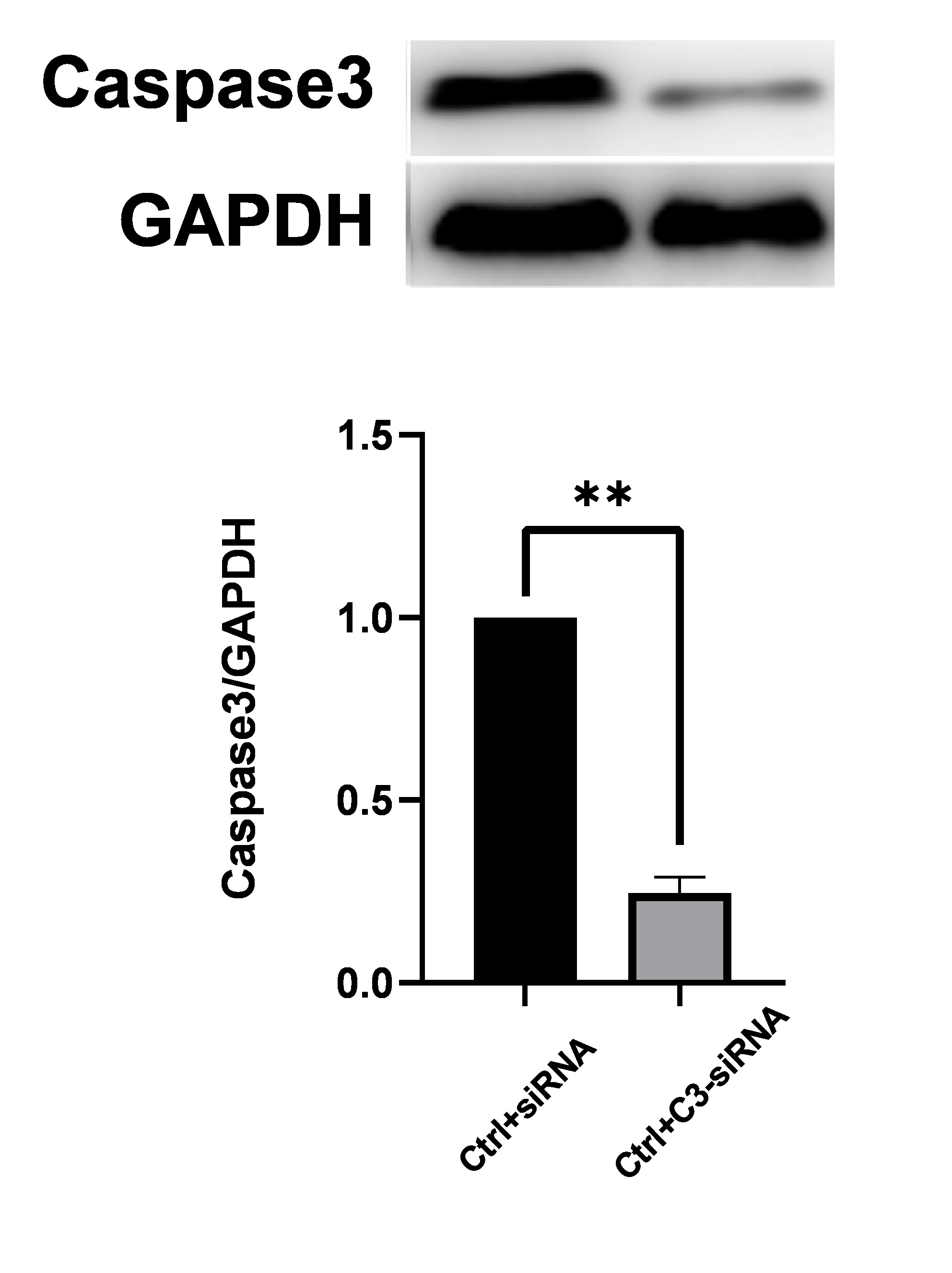

Supplement: Supplementary file 10 [file Image12.JPEG]

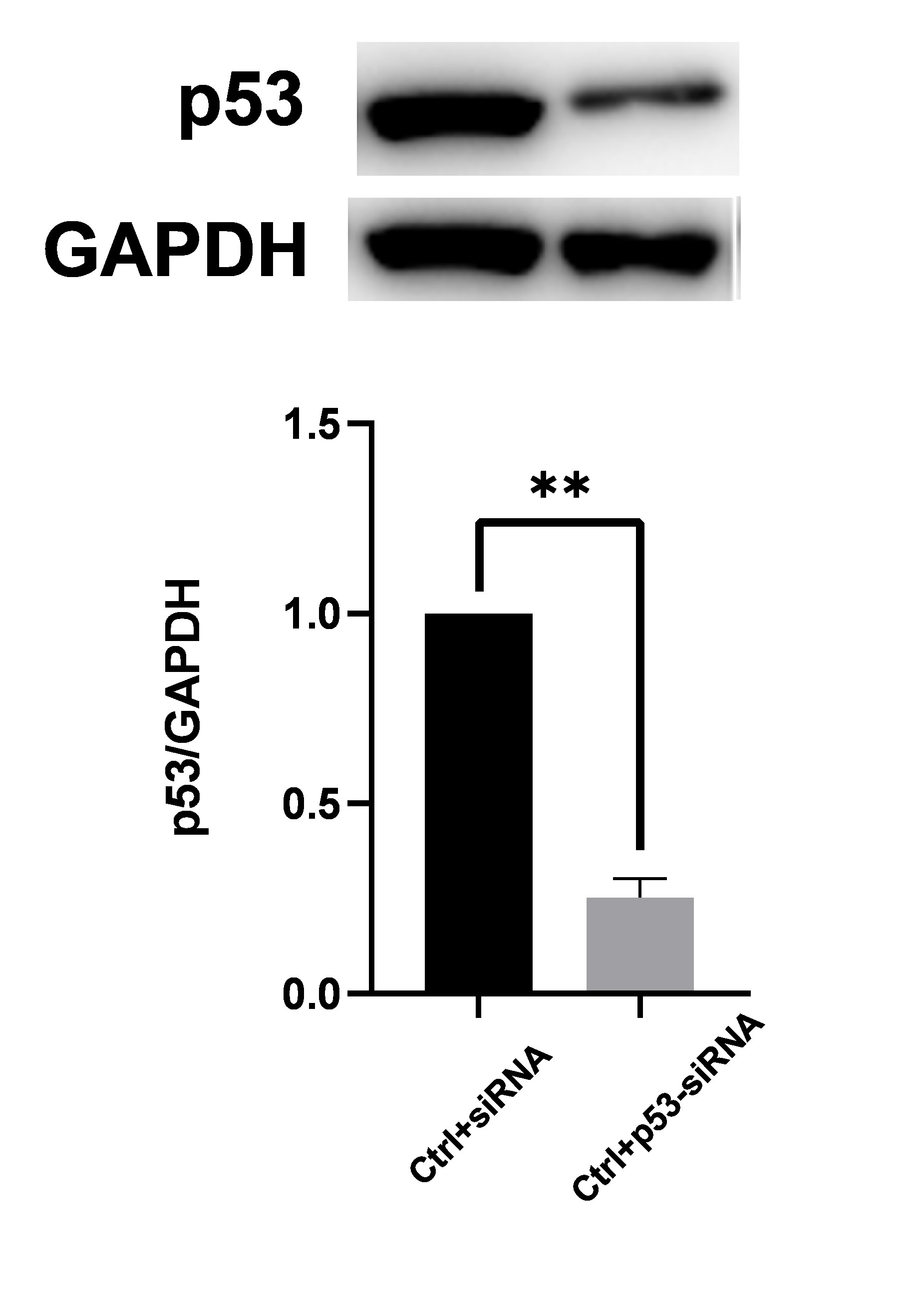

Supplement: Supplementary file 11 [file Image11.JPEG]

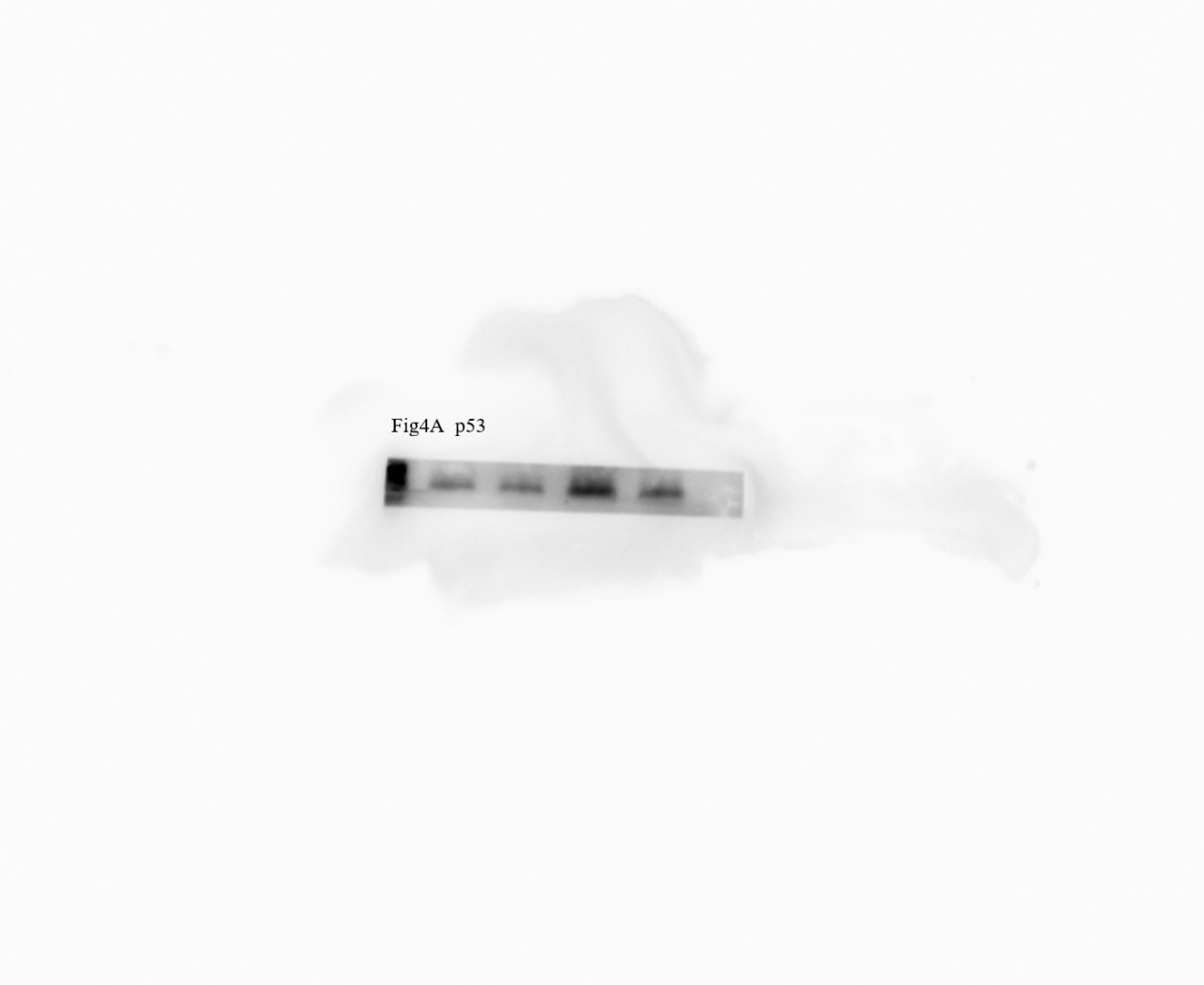

Supplement: Supplementary file 12 [file Image8.JPEG]

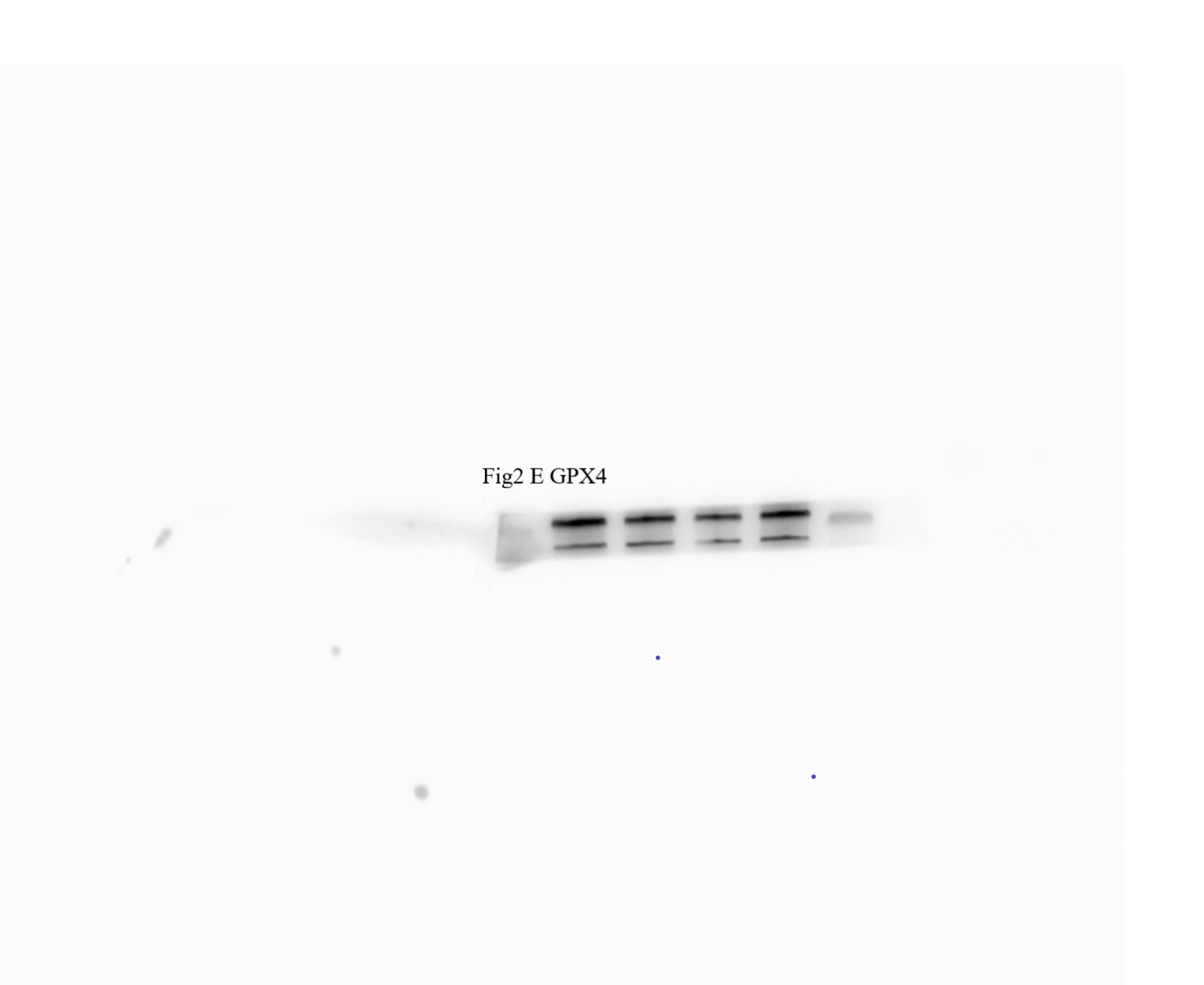

Supplement: Supplementary file 13 [file Image6.JPEG]
